# Mimicking the Effects of Antimicrobial Blue Light: Exploring Single Stressors and Their Impact on Microbial Growth

**DOI:** 10.3390/antiox13121583

**Published:** 2024-12-23

**Authors:** Beata Kruszewska-Naczk, Mariusz Grinholc, Aleksandra Rapacka-Zdonczyk

**Affiliations:** 1 Laboratory of Photobiology and Molecular Diagnostics, Intercollegiate Faculty of Biotechnology, University of Gdansk and Medical University of Gdansk, Abrahama 58, 80-307 Gdansk, Poland; beata.kruszewska@phdstud.ug.edu.pl (B.K.-N.); mariusz.grinholc@biotech.ug.edu.pl (M.G.); 2 Department of Pharmaceutical Microbiology, The Faculty of Pharmacy, Medical University of Gdansk, Hallera 107, 80-416 Gdansk, Poland

**Keywords:** antimicrobial blue light (aBL), *Escherichia coli*, hydrogen peroxide, hydroxyl radicals, Keio collection, oxidative stress, superoxide anion, single stressors

## Abstract

Antimicrobial blue light (aBL) has become a promising non-invasive method that uses visible light, typically within the 405–470 nm wavelength range, to efficiently inactivate a wide variety of pathogens. However, the mechanism of antimicrobial blue light (aBL) has not been fully understood. In this study, our research group investigated the sensitivity of *Escherichia coli* BW25113 single-gene deletion mutants to individual stressors generated by aBL. Sixty-four aBL-sensitive mutants were tested under conditions mimicking the stress generated by irradiation with aBL, with their growth defects compared to the wild-type strain. Results revealed no positive correlation between aBL and single stressors, indicating that aBL’s effectiveness is due to the simultaneous generation of multiple stressors. This multifactorial effect suggests that aBL targets microbial cells more precisely than single stressors such as hydrogen peroxide. No single gene knockout conferred specific resistance, highlighting aBL’s potential as an antimicrobial strategy.

## 1. Introduction

Antibiotic resistance is one of the most serious and frequent threats that the modern community has to face. According to the WHO Bacterial Priority Pathogens List (2024 update), critical pathogens, due to limited treatment options and high mortality rates, are carbapenem-resistant *Acinetobacter baumannii* (CRAB) and Enterobacterales (CRE), and also third-generation cephalosporin-resistant Enterobacterales (3GCRE). Since the previous report in 2017, no new, effective antibiotic treatment has been developed to combat infections caused by these pathogens, prompting the search for innovative strategies to fight bacterial infections [1]. One of the most promising strategies is the use of antibacterial blue light (aBL), which has been repeatedly proven to be effective in fighting antibiotic-resistant pathogens [2,3]. aBL treatment has emerged as a promising non-invasive approach that utilizes visible light, typically in the 405–470 nm range, to effectively inactivate a broad spectrum of pathogens. Unlike traditional antibiotics, aBL offers a selective mechanism that minimizes host tissue damage while targeting microbial cells. The efficacy of aBL is due to its ability to generate reactive oxygen species (ROS) within bacterial cells, due to the excitation of photosensitizing endogenous chromophores, such as flavins and porphyrins [4,5]. Upon the absorption of photon, these molecules generate singlet oxygen (^1^O_2_), hydrogen peroxide (H_2_O_2_), superoxide anions (O_2_^•−^), and hydroxyl radicals (•OH), which cause oxidative stress, leading to cell damage and death [5,6,7]. This mechanism of action is effective against both Gram-negative and Gram-positive bacteria, including antibiotic-resistant strains, making aBL an attractive alternative to conventional antimicrobials [8,9]. This study aimed to check the sensitivity of single-gene deletion mutants of *Escherichia coli* (*E. coli*) BW25113 [10,11] to single stressors generated by aBL to elucidate the aBL protective role of each aBL-hypersensitive gene, which may contribute to explaining the aBL mode of action. Sixty-four-aBL sensitive mutants were tested in conditions mimicking single species stress generated by aBL. Mutants’ hypersensitivity against individual stressors connected with aBL was tested measuring the growth defect compared to the wild-type strain, following a methodology analogous to that used in studies of nonthermal plasma [12].

## 2. Materials and Methods

### 2.1. Bacterial Strains

The analysis utilized a set of 64 *E. coli* BW25113 single-gene knockout mutants, (known as the Keio collection, consisting of 3985 strains as described by Baba et al., 2006 [10]) which were previously identified as aBL hypersensitive [11]. The list of aBL hypersensitive mutants used in this study includes the following: *atpA*, *atpB*, *atpC*, *atpD*, *atpE*, *atpF*, *atpG*, *atpH*, *cpxA*, *cydD*, *dacA*, *deoB*, *dnaJ*, *dnaK*, *ecnB*, *fabH*, *fimB*, *gmhB*, *gntK*, *hldD*, *holD*, *metR*, *narL*, *nuoN*, *oxyR*, *pfkA*, *pgi*, *pgm*, *phoQ*, *ppc*, *priA*, *purA*, *pyrE*, *rbfA*, *rfaC*, *rfaE*, *rfaG*, *rnt*, *rpe*, *sstT*, *surA*, *thyA*, *tolA*, *tpiA*, *truA*, *ubiC*, *umuD*, *ybaP*, *yccM*, *ydcE*, *ydcX*, *ydeU*, *yegS*, *yfbB*, *yfeH*, *yfgL*, *ygfZ*, *yheM*, *yhhH*, *yigL*, *yihE*, *yjeK*, *yncA*, and *ypjD.* All of them were described in detail in our previous research [11]. The wild-type strain *E. coli* BW25113 was used as a control. Table S4 lists the aBL-hypersensitive mutants with a description of the protein encoded by the deleted gene.

Single-gene mutants were cultured in LB medium (Carl Roth, Karlsruhe, Germany) containing 15 µM of kanamycin (Gibco, Zawroty, Poland). The wild-type strain was cultured in the same medium without antibiotics. Overnight cultures were prepared in an orbital incubator (Innova 40, Brunswick, Germany) at 37 °C, 150 rpm for 16–20 h.

### 2.2. Reagents

Paraquat and K_3_PO_4_ were purchased from Sigma Aldrich (Darmstadt, Germany), Triton X—Serva Electrophoresis (Heidelberg, Germany), H_2_O_2_—Pol-Aura (Zawroty, Poland), CuCl_2_—Thermofisher Scientific (Waltham, MA USA), Sodium Nitroprusside, and HCl—CHEMPUR (Piekary Śląskie, Poland).

### 2.3. Determining aBL Sensitivity

Overnight cultures of mutants were diluted to obtain 0.5 McF. A total volume of aliquots which was transferred to 96-microliter plates was 100 µL. The samples were irradiated with 43.2 J/cm^2^ of LED blue light (λmax 415 nm, irradiance 25 mW/cm^2^) (Cezos, Gdynia, Poland) according to previously described studies [11]. Then, 10 µL of aliquots was collected and streaked horizontally onto LB-agar plates (A&A, Gdansk, Poland) to assess growth reductions. Plates were incubated at 37 °C overnight, and surviving colonies were counted. Experiments were performed in triplicate.

### 2.4. Screening Against Defined Stressors

**Figure 1:**
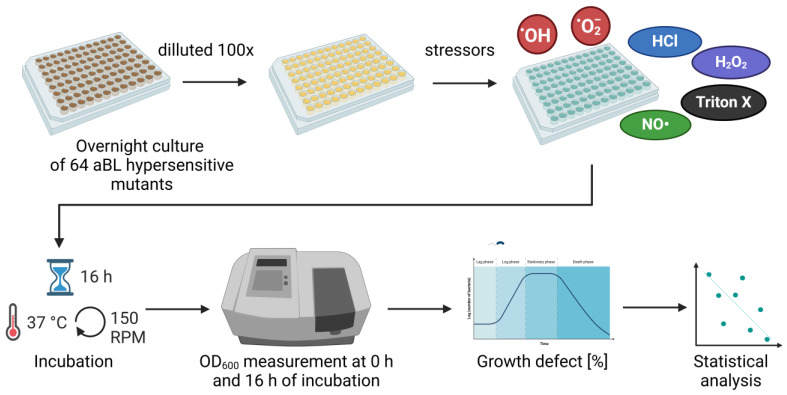
The experimental workflow.

The workflow is presented in Figure 1.

Overnight cultures of single-gene mutants of *E. coli* BW25113 [10] incubated at 37 °C by 16–20 h under aerobic conditions in an orbital incubator (Innova 40, Brunswick, Germany) were 100-fold diluted in LB in 96-well plates, and stressors were added. A medium containing the stressor at the appropriate concentration, determined as the dose, reduced the growth of the WT strain by 30–40%. A similar range of growth reduction was previously used in a study conducted by Krewing et al. (2019) [12] examining single plasma stressors. Concentrations of stressors are listed in Table 1. Reagents to investigate Fenton reaction products were prepared in a sterile LB medium buffered with K_3_PO_4_, pH = 6. After sterilization, CuCl_2_ and H_2_O_2_ were freshly added before experiments.

The OD_600_ value of bacteria with stressors and controls without stressors were measured in an EnVision Multilabel Plate Reader (PerkinElmer, Waltham, MA, USA) at 0 h and after 16 h of incubation at 37 °C with shaking (150 rpm). All the experiments were performed in five biological replicates.

∆OD_600_ values (OD_600_ 16 h − OD_600_ 0 h) were determined, and the sensitivity of each mutant was calculated according to the formula:

**grown defect [%]** = 100 − 100 × ∆OD_WT_ × ∆OD^−1^ _WT, stressed_ × (∆OD _mutant_ × ∆OD − 1 _mutant, stressed_)^−1^

The greater the growth defect value, the higher the sensitivity of the single-gene mutant to particular stress conditions. Strains with a growth defect of 20% or higher were termed ‘sensitive’ towards that stressor.

### 2.5. Statistical Analysis

The mean and standard deviation are used to describe continuous variables. For all statistical tests, a significance level of α = 0.05 was assumed. Due to the distribution of variables deviating from the normal distribution, the Spearman correlation coefficient with its statistical significance was calculated to investigate the correlation between aBL and stressors.

To calculate the probability that a mutant sensitive to a given stressor is also sensitive to another stressor, the formula for conditional probability was used:

$$P\left(A|B\right)=\frac{P\left(A\cap B\right)}{P\left(B\right)}$$

Explanation of the formula: P(A|B)—the probability that mutants A and B are simultaneously sensitive to a given stressor (in both cases, the average of 5 measurements is >20 or < −20). P(B)—the probability that the mutant is sensitive to stressor B.

Cluster analysis was performed to group mutants into groups with a similar stressor sensitivity profile. The analysis began by reducing the dimension of the data by using a Principal Component Analysis (PCA) performed on scaled data.

For the proper cluster analysis, the k-means++ method with cosine distances was used. The number of clusters was selected based on the WSS (Within-Cluster Sum of Squares) index and the Silhouette statistics. The Mann–Whitney U test was used to characterize the clusters to check the differences between stressors in different clusters. Due to multiple comparisons, the *p*-value was adjusted by the FDR (False Discovery Rate).

Protein–protein functional interaction networks and gene co-expression within clusters were analyzed using the STRING database. The analysis was performed using the data from the curated databases that were experimentally determined. 

## 3. Results

### 3.1. Growth Defects of aBL Hypersensitive Mutants Exposed to a Single Stressor

Single-gene mutants’ growth was affected by stressors in various ways, depending on the kind of stressor used. Table 2 presents growth defect values calculated according to the formula described in the Material and Methods Section, taking into account the growth of mutants without stressors and comparing it to the wild-type growth defect.

The mean and standard deviation for each stressor are provided in Table S1 in the Supplementary Materials.

### 3.2. Correlation Between aBL and Stressors

Table 3 shows the ρ-Spearman correlation coefficient (due to the data distribution being different from the normal distribution) along with the result of the test verifying its significance. This coefficient was calculated between aBL and each stressor. Based on the results, the following can be summarized:

- There is a negative, statistically significant correlation between aBL and H_2_O_2_ (ρ = −0.45, *p* < 0.001).
- There is a negative, statistically significant correlation between aBL and membrane stress (ρ = −0.31, *p* < 0.05).

For other stressors, no significant correlation was observed.

**Figure 2:**
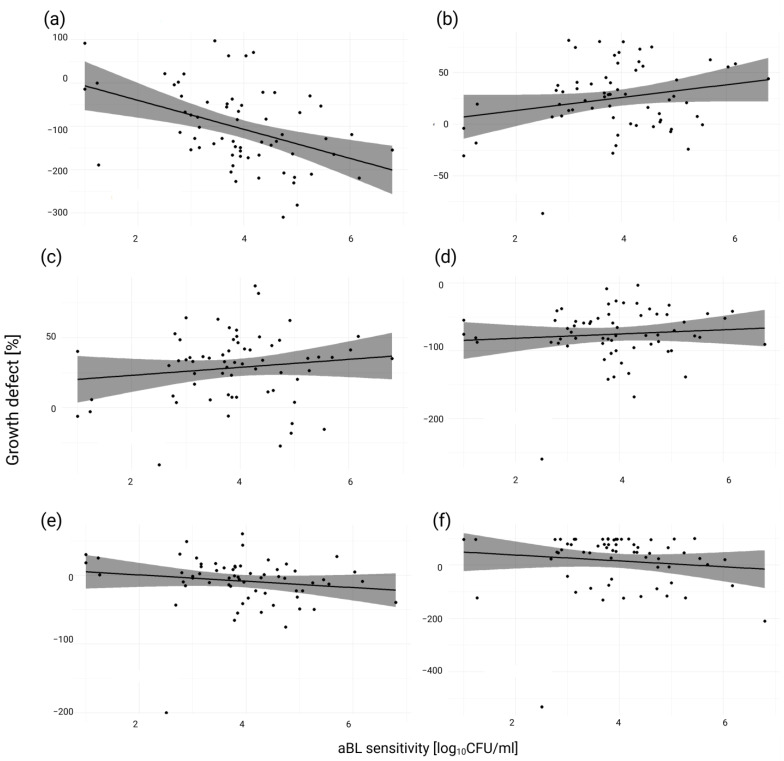
Correlograms illustrate the correlation between aBL and stressors: (**a**) H_2_O_2_, (**b**) O_2_^−^, (**c**) NO•, (**d**) Acidic pH, (**e**) membrane stress, and (**f**) •OH. The X-axis represents aBL sensitivity values [log_10_ CFU/mL], while the Y-axis growth defect [%] for each stressor.

Figure 2 presents correlation test results between aBL and stressors in the form of correlograms.

### 3.3. Evaluation of the Probability That a Mutant That Is Sensitive to One Stressor Is Sensitive to Another Stressor

In the next step, the conditional probability was calculated to check how the probability is distributed such that the mutant will be sensitive to stressor A, provided that it is also sensitive to stressor B. Results are presented in Table 4.

Based on the data obtained, the following can be observed:

- Mutants sensitive to H_2_O_2_ are also sensitive to acidic pH and •OH with more than 90% probability and to O_2_^−^ and NO• with more than 60% probability.
- Mutants sensitive to O_2_^−^ are also sensitive to H_2_O_2_, NO•, acidic pH, and •OH with more than 90% probability.
- NO• sensitive mutants are also sensitive to H_2_O_2_, acidic pH, and •OH with more than 90% probability and to O_2_^−^ with more than 75% probability.
- Mutants sensitive to acidic pH are sensitive to H_2_O_2_ and •OH with more than 90% probability, and to O_2_^−^ and NO• with more than 60% probability.
- Mutants sensitive to membrane stress are also sensitive to H_2_O_2_ with a probability of 0.88, to acidic pH and •OH with a probability greater than 80%, and to O_2_^−^ and NO• with a probability of more than 50%.
- Mutants sensitive to •OH are also sensitive to H_2_O_2_ and acidic pH with more than 90% probability and to O_2_^−^ and NO• with more than 65% probability.

Detailed analysis is presented in Supplementary Materials (Tables S2 and S3; Figures S1 and S2).

### 3.4. Cluster Analysis

To group mutants into groups with a similar stressor sensitivity profile, cluster analysis was performed. Because the data table has six different columns, there was a need to reduce the data dimension before the actual analysis, i.e., create two such variables that will represent the six source columns. To achieve this, a Principal Component Analysis (PCA) was performed.

Before the PCA procedure began, the data were scaled so that their mean and standard deviation were 0 and 1, respectively. Because this analysis is very sensitive to outliers, the mutant *ygfZ* was removed from the analysis, whose values were significantly different from the rest. The data have also been centered around 0 to remove constants from the data that add nothing to the knowledge of data variation.

**Figure 3:**
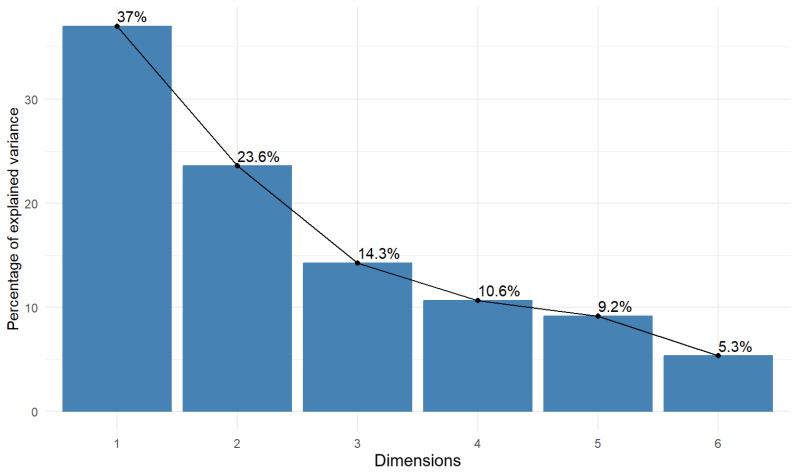
Scree plot. The X-axis shows the number of dimensions, while the Y-axis shows the percentage of variance explained by each dimension. The scree point is observed where variance starts to level off after the second dimension.

To select the number of dimensions, a landfill chart was made based on which such a selection can be made. This graph is shown in Figure 3.

In Figure 3, two dimensions (components) “explain” 60.6% of the variability.

Table 5 shows the factor loadings and the coefficient of determination (factor loading squared) for each component. Factor loads measure the correlation between a given component and a source variable, i.e., they represent the influence of individual variables on a given principal component. In the results of our analysis, factor loadings measure the variability of six stressors and show how strongly each stressor is connected with the given component. Factors loadings for particular stressors whose values are closer to 0 are more weakly correlated with the given components than values close to 1 and −1. The coefficient of determination R^2^ shows the percentage of the variability of a given component that is explained by a given input variable.

For further analysis, the first two components were considered, the first of which explains 37% of the variability of the output variables, and the second 23.6%, so, together, they explain more than 60% of the variability of the output data. All factor loadings for PC1 are positive, which means that, as PC1 increases, the stressor values will also increase. In the case of PC2, this will be the case for H_2_O_2_. For membrane stress and •OH, the opposite situation is true, i.e., as PC2 increases, the stressor values will decrease.

In the next step, cosine distances were calculated for the data from both main components, necessary for further analysis.

**Figure 4:**
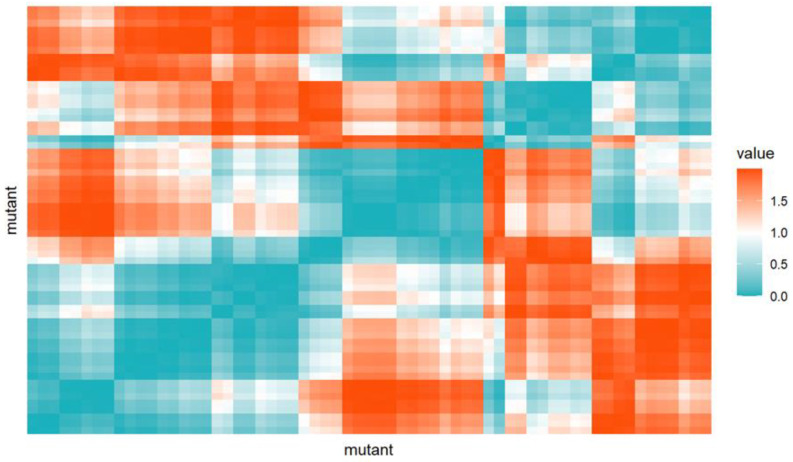
Cosine distances heatmap: On the X-axis, mutants are listed from left to right in order: *tolA*, *pfkA*, *yhhH*, *narL*, *rpe*, *metR*, *deoB*, *rnt*, *oxyR*, *nuoN*, *cydD*, *holD*, *atpC*, *thyA*, *dacA*, *pgi*, *pgm*, *atpG*, *truA*, *phoQ*, *umuD*, *gmhB*, *rfaD*, *tpiA*, *yihE*, *rfaE*, *rfaG*, *atpE*, *yfgL*, *ybaP*, *atpB*, *atpH*, *dnaJ*, *yfbB*, *priA*, *rbfA*, *ubiC*, *yegS*, *atpF*, *rfaC*, *cpxA*, *yccM*, *ppc*, *yjeK*, *pyrE*, *sstT*, *dnaK*, *yheM*, *ecnB*, *ydcX*, *atpD*, *ydcE*, *atpA*, ypjD, *fabH*, *surA*, *purA*, *fimB*, *ydeU*, *yigL*, *gntK*, *yfeH*, *yncA*. The Y-axis mutants are presented in the opposite order than on the X-axis.

The first of such analyses is the creation of a distance map, in which each pair of mutants is assigned a color according to their previously calculated distance. In this way, it is possible to initially draw attention to potential groups of similar mutants. Because a given map is symmetrical along the diagonal, one of them is paid attention to when interpreting. The cosine distance heatmap is presented in Figure 4.

**Figure 5:**
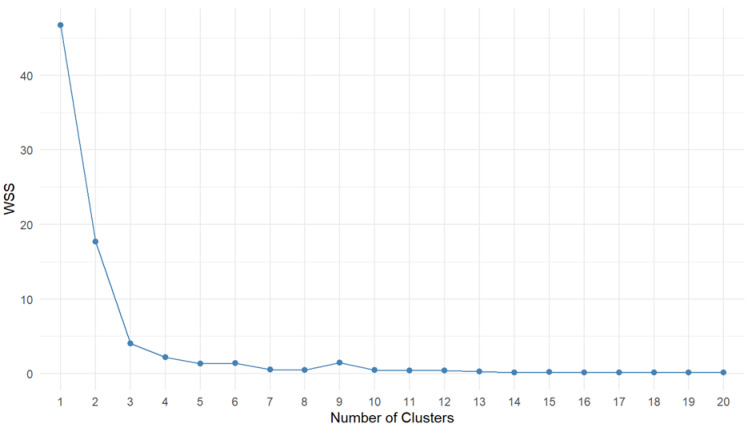
The WSS plot for determining the number of clusters. WSS—Within-Cluster Sum of Squares. The number of 3 clusters was chosen as the optimal value.

In the next step, the clustering method was selected. The k-means++ method was chosen, so the appropriate number of clusters must be selected in advance. One method is to select the number of clusters based on the WSS (Within-Cluster Sum of Squares) ratio (Figure 5).

The optimal number of clusters is the one at which the WSS value does not increase significantly after increasing the number of clusters. In this case, looking at Figure 5, we conjectured that the optimal number of clusters is equal to three. To verify this, another measure—the Silhouette statistic—was used, which measures how well each of the observations fits into the cluster assigned to it. A value close to one means that the observation is placed very close to the center of the cluster, and a statistic close to zero means that the observation lies on the border of two clusters. Negative values indicate that the value is potentially matched to the wrong cluster.

**Figure 6:**
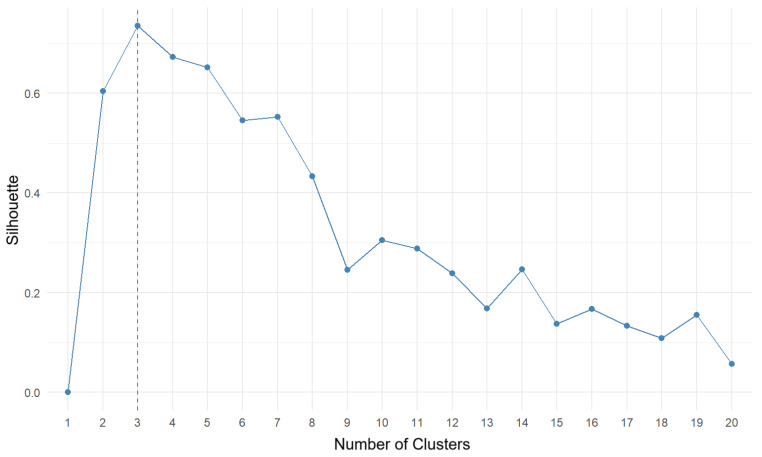
Silhouette plot for determining the number of clusters. The Y-axis shows the silhouette score for each cluster shown on the X-axis.

**Figure 7:**
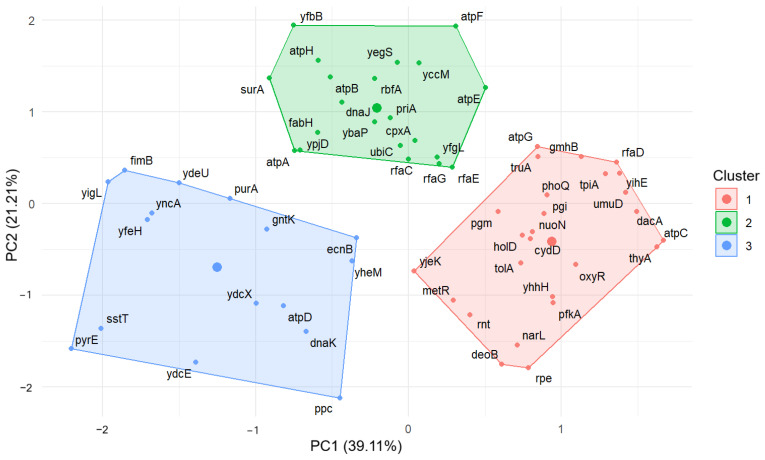
Results of k-means++ algorithm for both principal components (PC1—X-axis and PC2—Y-axis) summarizing clustering analysis. Bolded dots in the center of each cluster represent their centroids placed at the longest distance from other clusters.

The silhouette plot (Figure 6) confirmed that the optimal number of clusters is equal to three. Based on the above, the division of the data into three clusters was chosen. Figure 7 shows the final division of data into three clusters using the k-means++ algorithm.

**Figure 8:**
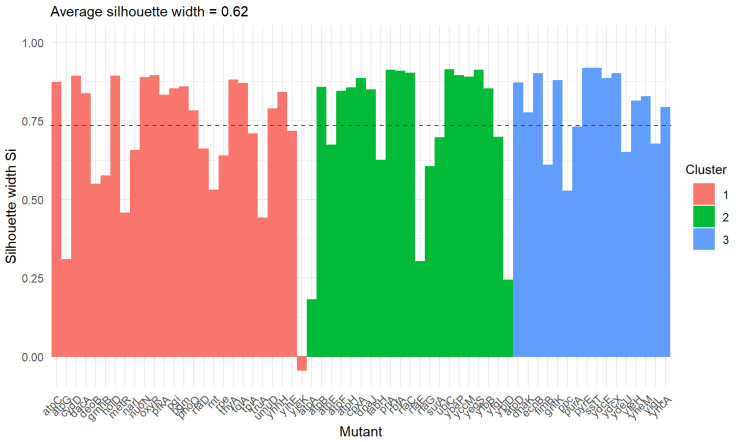
Cluster silhouette plot representing the silhouette width for each mutant in every cluster. The red line indicates the average value for the entire set.

Based on the statistical analysis, mutants were grouped into three clusters, and their characteristics are presented below. Figure 8 shows the silhouette stat values for each point (mutant). The horizontal red line is at the height of the average value of the statistic for the entire set.

The value for the mutant *yjeK* is below 0. Such a value can be interpreted as a potentially incorrectly assigned value, but it is worth remembering that this criterion refers to the distance of a given point from the center of the cluster (marked with a slightly larger point than the others).

The list of mutants assigned to one of three clusters is summarized in Table 6. The genes assigned to cluster 1 are involved in the key cellular processes such as energy metabolism and core cellular processes (*atpC*, *atpG*, *nuoN*, *pfkA*, *pgi*, *pgm*, *rpe*, *tpiA*, *deoB*), oxidative stress response and adaptation (*oxyR*), regulation and adaptation (*phoQ*, *metR*), membrane integrity and structure (*tolA*, *rfaD*), DNA replication and repair (*thyA*, *umuD*), and translation (*truA*, *rnt*). The genes assigned to cluster 2 are involved in processes such as energy generation (*atpA*, *atpB*, *atpE*, *atpF*, *atpH*), stress response and environmental adaptation (*cpxA*, *dnaJ*), and fatty acid biosynthesis (*fabH)*. Cluster 3 comprises genes involved in stress response (*dnaK*, *ecnB*), metabolism (*gntK*, *ppc*, *purA*, *sstT)*, and DNA repair and genome stability (*ydcE*, *ydcX*, and *pyrE*) under conditions of stress or damage.

Table 7 provides information about the median and interquartile range of growth defects for each cluster and each stressor. Univariate tests were also performed comparing these medians between each pair of clusters. To perform this, the Mann–Whitney U test was performed, which checks the statistical significance between the two medians (the median comparison test was chosen due to the lack of normality of the data distribution). As multiple comparisons were made, a *p*-value adjustment (FDR) was applied.

**Figure 9:**
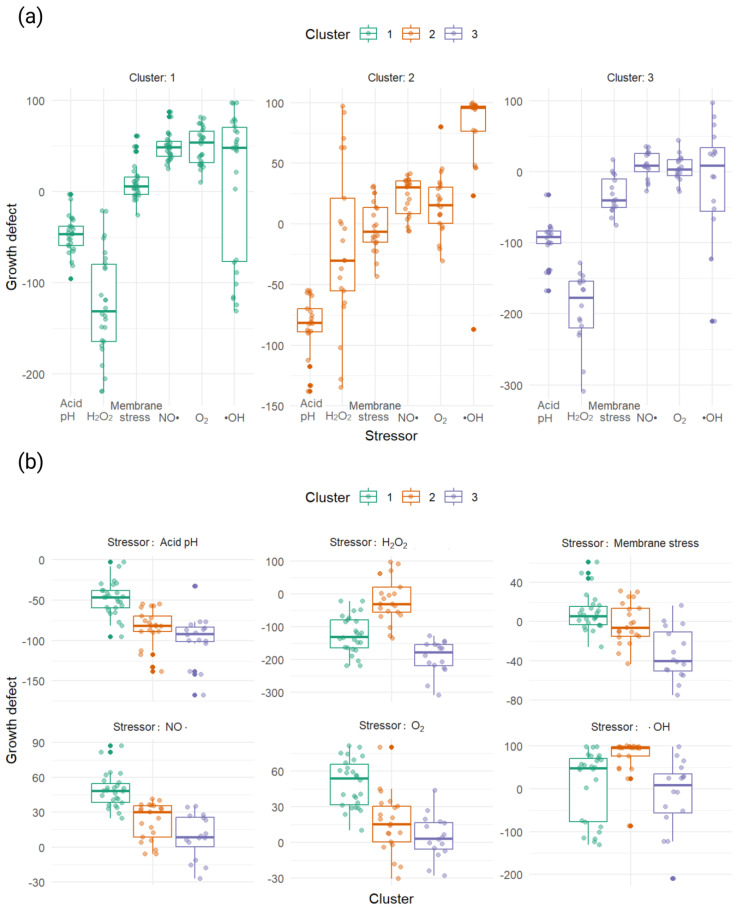
Growth defects of mutants by clusters: (**a**) characterization of growth defect profiles for 3 clusters; and (**b**) characterization of cluster profiles for each stressor.

Data from Table 7 are also presented in Figure 9 and Figures S3 and S4 in Supplementary Materials.

The analysis results demonstrated several key findings:

- When exposed to an acidic pH, the median growth defect for all mutants across the clusters was less than 0. However, the first cluster exhibited the highest median growth defect at −46.39 [−59.36; −38.08], which was statistically significantly higher than that of the other clusters. The medians of the second and third clusters did not differ significantly from each other, with values of −81.65 [−89.11; −70.02] for cluster 2 and −91.61 [−101.53; −83.61] for cluster 3.
- In the presence of H_2_O_2_, the median growth defect remained less than 0 in all clusters, with statistically significant differences observed between each pair of clusters. The second cluster showed the largest growth defect, at −177.62 [−219.96; −154.38], while the first cluster exhibited the smallest defect at −29.96 [−55.11; −21.01].
- When exposed to membrane stress, the median growth defect for the first cluster was positive (5.67 [−3.02, 15.68]), but it was not statistically significantly different from the second cluster, which had a median of −6.38 [−15.16, 13.71]. The third cluster, however, showed a statistically significantly smaller median defect (−40.22 [−50.5; −10.42]) compared to the other two clusters.
- Upon exposure to nitric oxide (NO•), the median growth defect for all clusters was greater than 0, with significant differences between each pair of clusters. The first cluster exhibited the largest growth defect (48.35 [38.55; 54.74]), whereas the second cluster had the smallest (30.29 [8.39; 35.42]).
- In response to O_2_⁻, the median growth defect was greater than 0 for all clusters. The first cluster showed the largest and most statistically significantly different median (53.98 [31.89; 65.82]), while the medians for the second and third clusters were not significantly different from each other.
- Lastly, when mutants were exposed to hydroxyl radicals (•OH), the median growth defect was again greater than 0 across all clusters. The second cluster exhibited the largest median defect (95.89 [76.47; 97.31]), which was statistically significantly different from the other two clusters, whose medians were not significantly different from each other.

**Figure 10:**
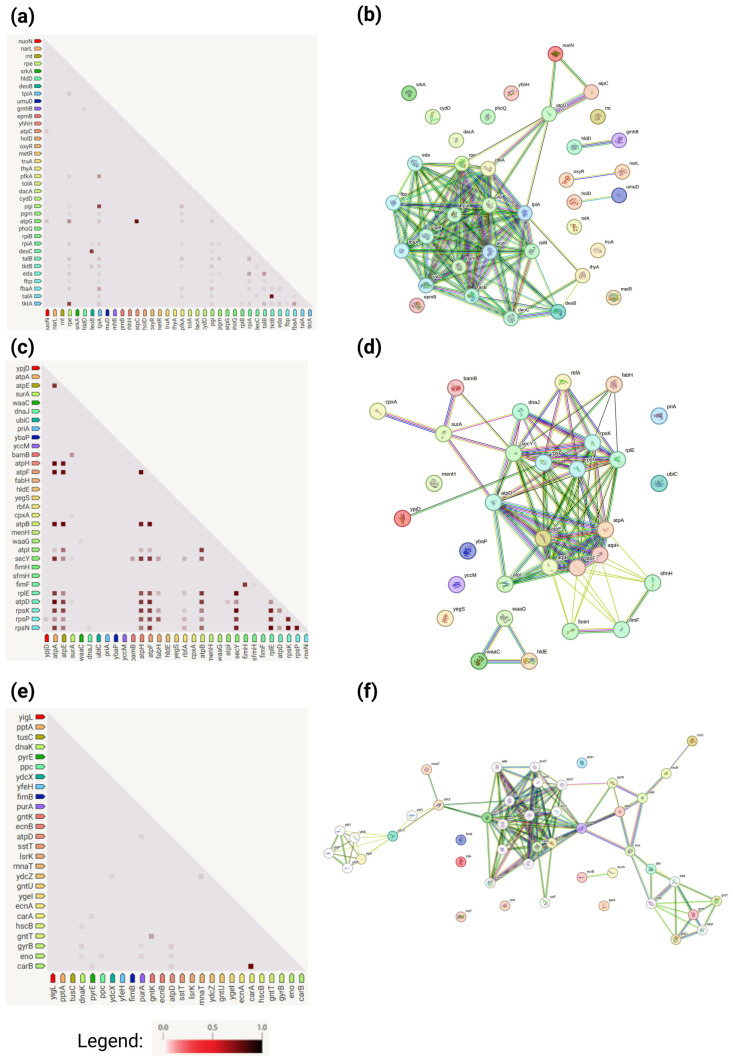
Protein–protein functional interaction networks and gene co-expression. Protein–protein functional interaction networks of the proteins are divided into 3 clusters: (**a**) Gene co-expression within cluster 1; (**b**) protein–protein functional interaction networks within cluster 1; (**c**) gene co-expression within cluster 2; (**d**) protein–protein functional interaction networks within cluster 2; (**e**) gene co-expression within cluster 3; (**f**) protein–protein functional interaction networks within cluster 3. The analysis was performed with the STRING database (https://string-db.org). The colors of the lines denote the following: light blue, interactions known from curated databases; pink, interactions experimentally determined; bright green, predicted reaction (gene neighborhood); red, gene fusions; dark blue, gene co-occurrence; green, textmining; black, co-expression; and blue, protein homology.

Protein–protein functional interaction networks and gene co-expression analysis performed with the STRING database (https://string-db.org, accessed on 28 October 2024) revealed that many genes within one cluster are related in some way (e.g., protein–protein interactions, gene neighborhood, gene fusions, gene co-occurrence, co-expression, protein homology). The data obtained from the STRING database are presented in Figure 10.

## 4. Discussion

Bacterial populations exhibit a remarkable ability to adapt to extreme environmental fluctuations. In some cases, adaptation to one stressful condition confers a fitness advantage when cells encounter a second stressor, a phenomenon referred to as cross-stress protection [13]. aBL has a multi-target mode of action, which is a key factor contributing to its efficacy against a wide range of microorganisms [8]. Unlike traditional antibiotics that often target one specific metabolic pathway or cellular component, aBL is based on generating ROS that simultaneously affect multiple cellular targets [7,14]. These ROS, including singlet oxygen (^1^O_2_) [7,15], superoxide anions (O_2_⁻) [5], hydroxyl radicals (•OH) [7], and hydrogen peroxide (H_2_O_2_) [5,6], are produced within the microbial cells during exposure to aBL and cause widespread oxidative damage [7]. This study aimed to assess the sensitivity of single-gene deletion mutants of *E. coli* BW25113 to individual stressors generated by aBL to elucidate the protective role of each gene associated with aBL hypersensitivity. A total of 64 aBL-sensitive mutants that had been identified in our previous study [11] were tested under conditions that simulated the specific, single stress factors produced by aBL. The hypersensitivity of the mutants to individual aBL-related stressors was evaluated by measuring their growth defects, in comparison to the wild-type strain. By isolating and assessing the effects of single stressors, we aimed to dissect the complex interplay between these reactive molecules and better understand their individual roles in microbial inactivation. Studying single stressors, such as H_2_O_2_, with selected mutants provides a clearer picture of how bacteria respond to targeted oxidative stress. This study also clarifies that the combination of stressors generated by aBL is often more effective than any single factor alone. Furthermore, understanding how different mutants respond differently to individual stressors compared to aBL as a whole may reveal vulnerabilities in microbial defense mechanisms that could be exploited in the clinical settings in the future.

Hydrogen peroxide is a widely used antimicrobial agent that generates ROS directly upon application [16,17,18]. When H_2_O_2_ comes in contact with microbial cells, it can be broken down into hydroxyl radicals and other ROS via Fenton reactions or catalase activity [19]. These ROS attack cellular structures, including DNA [20]. While both aBL and H_2_O_2_ generate ROS, aBL relies on the internal activation of chromophores within the microorganisms [21], whereas H_2_O_2_ is an external oxidative agent that can penetrate and damage cells on contact. aBL has the advantage of being non-toxic to mammalian tissues [22], avoiding the issues of toxicity associated with H_2_O_2_ [23].

The superoxide anion (O_2_⁻) is highly reactive and toxic to microbial cells. Once generated, it can initiate a cascade of oxidative reactions that damage essential cellular components, such as lipid peroxidation, DNA and protein damage, and the disruption of cellular enzymes [24,25]. It can also lead to secondary ROS production: O_2_⁻ can be converted into other ROS, such as H_2_O_2_ and •OH, through enzymatic or non-enzymatic dismutation reactions. For instance, superoxide dismutase (SOD) in microbial cells can convert O_2_⁻ into H_2_O_2_ [26,27].

The highly reactive hydroxyl radicals (•OH) can be generated through the Fenton reaction, where hydrogen peroxide (H_2_O_2_) reacts with transition metals like iron (Fe^2+^). This process converts H_2_O_2_ into hydroxyl radicals [28]:

H_2_O_2_ + Fe^2+^→Fe^3+^ + •OH + OH⁻.

Hydroxyl radicals (•OH) are one of the most destructive forms of ROS that cause extensive damage to essential microbial cellular components [29]. Because hydroxyl radicals react almost immediately with any biological molecule they encounter, their antimicrobial effect is rapid and highly destructive [25]. The non-specific nature of •OH means that it can attack a wide variety of targets within microbial cells, making it a critical player in the cell-killing process. It is assumed that these radicals (along with singlet oxygen) have a broad spectrum of antimicrobial activity of aBL [7,30].

Nitric oxide (NO•) is a type of reactive nitrogen species (RNS) known for its antimicrobial properties. NO• production is often associated with immune responses in mammalian cells [31]. It is not typically produced as a primary reactive species during aBL irradiation; however, combining aBL with nitric oxide donors could provide a synergistic antimicrobial effect [32,33]. The combination of NO• and ROS from aBL could enhance oxidative and nitrosative stress in microbes. Moreover, Liebmann et al. (2010) showed that blue light up to a wavelength of 453 nm is capable of releasing nitric oxide from nitrosated proteins and that NO• can initiate cell differentiation in human skin [34].

The pH of the environment can play a significant role in the effectiveness of aBL, especially if the environment is acidic [35]. Acidic pH could promote the accumulation of intracellular ROS [36]. In clinical applications, understanding the pH of the infection site can increase the treatment effectiveness [37,38,39]. For example, infections associated with the elevated alkaline milieu, such as chronic wounds [40], may require adjustments of aBL protocols to maximize efficiency.

In our study, we also mimicked the membrane stress, because aBL induces significant membrane stress, due to lipid peroxidation, which disrupts the bacterial cell membrane, increasing permeability and compromising cellular integrity [4].

Similar studies have been conducted in the context of plasma by Krewing et al. (2019). The researchers exposed mutant strains of E. coli to stressors mimicking plasma species, such as H_2_O_2_, O_2_⁻ and NO•, to analyze growth defects in the single-gene mutants. The results revealed that H_2_O_2_, O_2_⁻, and NO• showed the highest severity scores, suggesting that the absence of the protective genes makes the cells particularly vulnerable to plasma exposure [12].

This research presents that (excluding singlet oxygen, which was not studied here) the most significant single stressor in the bacterial response to aBL could be •OH and O_2_ because the highest number of aBL-hypersensitive mutants (45) treated with these stressors has a defect of growth greater than 20% compared to the wild-type strain (Table 7). The next stressors that affected mutant growth the most were NO• (36), membrane stress (9), and H_2_O_2_ (7). No mutant’s growth was affected by acidic pH, which may indicate that acidic pH, when applied as a single stressor, does not significantly affect the growth of E. coli mutants; however, it is known that acidic pH plays a role in the bacterial response to aBL [35]. While acidic pH alone is not enough to cause growth inhibition, it could sensitize the bacteria to oxidative stress from ROS induced during aBL exposure.

The observed discrepancies in sensitivity between stressor A and stressor B (and vice versa) (Table 4) may be caused by both the substitution of data into the conditional probability formula and also by the different biological effects induced by different stressors.

Mathematically, the formula P(A|B) = P(A∩B)/P(B) inherently depends on the distribution of sensitive mutants in the data set. For example, if the mutants sensitive to stressor B constitute a smaller subset, the probability P(B) in the denominator decreases the overall conditional probability. Conversely, if a larger number of mutants are sensitive to stressor A, this increases the numerator (P(A∩B)), leading to higher probabilities in one direction compared to the other. Biologically, differences in the reactivity and mechanisms of action of the stressors also play a significant role. For example, H_2_O_2_ and O_2_^−^ differ in their redox potential and target specificity within the cell, which may lead to varying degrees of overlap in sensitivity between mutants. Moreover, these two ROS forms activate different oxidative stress response pathways in bacteria cells [41]. Mutants that are sensitive to H_2_O_2_ may exhibit cross-sensitivity to O_2_^−^, but the inverse may not hold true due to differences in their cellular pathways and stress responses.

Mutants sensitive to four stressors were as follows: *atpC*, *gmhB*, *rfaD*, and *yccM*. *atpC* encodes a subunit of the ATP synthase complex. ATP synthase plays a crucial role in cellular energy metabolism, particularly when energy production is challenged. For instance, a rapid increase in ATP could be observed after DNA damage [42]. It is probable that, when cells face oxidative stress, they require efficient ATP production to activate stress-response mechanisms and repair oxidative damage. Defects in ATP synthase function can impair the ability of *E. coli* to cope with oxidative stress. *gmhB* and *rfaD* are genes involved in the lipopolysaccharide (LPS) biosynthesis pathway. Mutations in LPS biosynthesis genes could increase membrane permeability, making cells more vulnerable to environmental stresses (such as oxidative stress). A study conducted by Seregina et al. (2022) revealed that the inactivation of *gmhB* and *rfaD* led to dramatic changes in the redox status of cells [43]. Disruptions in LPS assembly, like those caused by *gmhB* and *rfaD* mutations, can lead to heightened sensitivity to ROS and other stressors. Mutants unaffected by any stressors were *atpD* and *ydcX* probably because they respond only to the combination of stressors, or the growth defect can be caused by other aBL-generated stressors (for example, singlet oxygen not tested in this study).

Because no positive correlation between aBL and single stressors was found, we can conclude that the bactericidal role of aBL is a sum of the combination of stressors, not an effect of the single one. aBL produces a variety of ROS (^1^O_2_, O_2_⁻, •OH, H_2_O_2_) [7,44]. These ROS act together to cause oxidative stress and damage to microbial cells [45,46,47]. By treating bacterial cells with H_2_O_2_ or other single stressors alone, we could not observe additive or synergistic effects of these other ROS: the different types of ROS generated by aBL can interact with each other. For instance, superoxide anion (O_2_⁻) and H_2_O_2_ are relatively non-toxic per se but, via the Fenton reaction, they lead to hydroxyl radical (•OH) production, which is much more reactive and damaging [19,46,48]. By using only H_2_O_2_, perhaps not enough (if any) of the other important ROS were generated, which may explain the lack of positive correlation. What is no less important, during aBL, ROS are generated within the microbial cells, often near sensitive cellular components like the membranes, organelles, nucleic acids, or enzymes [19,47,49]. In contrast, adding H_2_O_2_ externally might not replicate this localized effect. H_2_O_2_ itself in an aqueous solution does not oxidatively modify DNA, lipids, or proteins in the absence of catalysts for radical formation [50,51]. H_2_O_2_ diffuses freely, and its action is more generalized, leading to less specific damage [52] compared to ROS released during aBL.

A selection of the optimal concentration of H_2_O_2_ was also complicated during this study. Too low a concentration may not cause significant oxidative damage, while too high a concentration could trigger bacterial defense mechanisms (e.g., catalase or peroxidase activity), reducing its impact [52]. Probably, aBL generates smaller, controlled amounts of H_2_O_2_ along with other ROS, potentially allowing for more sustained damage without triggering strong defense responses. aBL might overwhelm microbial defenses by generating ROS in bursts during irradiation, whereas H_2_O_2_ application as a single stressor might allow bacteria more time to respond and detoxify the environment [53,54,55]. When exposed to H_2_O_2_ alone, bacterial cells may upregulate protective enzymes more effectively compared to exposure to aBL. As a result, cells may be better armed to cope with H_2_O_2_ as a single stressor than with the mixed ROS profile generated by aBL. Moreover, H_2_O_2_ is less reactive compared to other ROS: H_2_O_2_ is relatively more stable than other ROS like singlet oxygen or hydroxyl radicals [19,46]. Therefore, when used as a single stressor, its impact might be weaker or slower. Singlet oxygen (^1^O_2_) and hydroxyl radicals (•OH) are much more reactive and can cause more immediate and severe damage to microbial components [56,57], which H_2_O_2_ alone probably cannot mimic.

Based on the results, the division of the data into three clusters was chosen. The key functional and biological significance across the clusters was summarized in the table below (Table 8).

Cluster 1 focuses on central metabolism and oxidative stress, integrating glycolysis, LPS biosynthesis, ROS protection, and nitrogen metabolism. It is also connected with significantly higher sensitivity to O_2_^−^ and NO• of this cluster in comparison to cluster 2 and 3.

Cluster 2 is strongly tied to energy generation needed for the activity of detoxification enzymes, membrane integrity, and adaptation to environmental stress. Mutants in this cluster are significantly more sensitive to H_2_O_2_ and •OH stressors in comparison to mutants from other clusters.

Meanwhile, cluster 3 emphasizes protein quality control, intermediary metabolism, and cellular nucleotide synthesis. These general functions do not play a crucial role in the tested bacterial stress response, as evidenced by the minimal growth defects observed in this cluster compared to others. Moreover, some cross-cluster functional integration may be observed: energy metabolism and stress response genes (*atp*, *dnaK*, *cpxA*) span clusters, indicating their centrality to bacterial survival. Furthermore, stress response regulators (*oxyR*, *cpxA*, *dnaJ*) link different metabolic pathways that may play a role in the coordinated adaptation process. Functional interaction networks and gene co-expression analysis performed with the STRING database present connections between genes and their collaboration in the particular stress response [58]. This also confirms that this process is multigene-regulated.

## 5. Conclusions

Because no positive correlation between aBL and single stressors was found, we can conclude that the effectiveness of aBL depends on the variety of stressors that are generated simultaneously during irradiation. When aBL is applied, the production of ROS occurs naturally within microbial cells. It probably makes the process highly targeted. This could distinguish aBL from single chemical stressors like hydrogen peroxide, which introduces ROS from an external source. The presented results indicate that aBL is multifactorial, and the bactericidal role of aBL is a sum of the combination of stressors, not an effect of the singular one. Moreover, no specific genes were detected, the knockout of which determines the development of a definite resistance to stressors. This suggests that aBL may be a promising strategy in the fight against pathogens.

## Figures and Tables

**Table 1:** Concentrations of stressors used in this study.

Stressor	Substance	Concentration
NO•	Sodium nitroprusside	0.5 mM
O_2_^−^	Paraquat	1 mM
Membrane stress	TritonX	0.125%
Acid stress	HCl	7 mM
H_2_O_2_		1 mM
•OH	CuCl_2_, H_2_O_2_	2.5 mM CuCl_2_ + 1.5 mM H_2_O_2_

**Table 2:** Growth defects [%] of aBL hypersensitive mutants exposed to single stressors compared with aBL sensitivity [log_10_ CFU/mL]. Calculations are performed for 5 biological replicates. Values with growth defects higher than 20% are bolded.

		Stressors					
Mutant	aBL Sensitivity	H_2_O_2_	O_2_^−^	NO•	Acidic pH	Membrane Stress	•OH
*atpA*	4.61	−134.65 (±37.8)	−2.2 (±10.43)	12.35 (±18.21)	−90.3 (±37.87)	−1.78 (±8.48)	**95.48 (±3.28)**
*atpB*	1.00	−13.9 (±149.98)	−4.03 (±45.42)	−6.06 (±41.91)	−75.59 (±26.4)	18.49 (±9.59)	**95.89 (±3.28)**
*atpC*	3.93	−168.96 (±36.7)	**59.31 (±20.35)**	**51 (±29.07)**	−26.43 (±19.82)	**43.94 (±8.45)**	**51.06 (±47.38)**
*atpD*	3.85	−227.06 (±51.25)	6.47 (±13.64)	7.63 (±34.86)	−84.74 (±25.35)	−2.32 (±26.9)	−52.43 (±24.94)
*atpE*	2.76	2.02 (±125.97)	**32.85 (±40.98)**	8.39 (±36.26)	−55.05 (±31.93)	**31.19 (±27.72)**	**95.97 (±1.91)**
*atpF*	1.00	91.81 (±3.33)	−30.66 (±30.57)	**40.24 (±23.59)**	−54.69 (±38.16)	**30.21 (±13.47)**	**95.89 (±3.79)**
*atpG*	3.96	−51.25 (±155.4)	**69.61 (±23.38)**	**46.51 (±20.24)**	−65.69 (±47.99)	−9.68 (±31.25)	**96.68 (±2.02)**
*atpH*	1.23	−0.02 (±129.74)	−18.11 (±31.97)	−2.82 (±36.1)	−80.81 (±40.86)	**25.36 (±12.23)**	**95.8 (±5.45)**
*cpxA*	3.16	−102.12 (±108.26)	**34.43 (±18.78)**	16.84 (±29.9)	−81.65 (±16)	12.67 (±12.29)	**97.31 (±2.04)**
*cydD*	4.28	−218.89 (±16.31)	**52.31 (±20.19)**	**87.11 (±8.58)**	−95.42 (±24.8)	−3.58 (±8.36)	**77.15 (±45.4)**
*dacA*	3.59	−128.05 (±50.37)	**80.23 (±6.83)**	**63.23 (±8.5)**	−52.23 (±8.09)	11.04 (±19.48)	**71.53 (±47.16)**
*deoB*	4.08	−172.58 (±43.14)	**29.26 (±27.82)**	**42.02 (±16.05)**	−29.15 (±38.28)	2.88 (±27.49)	−124 (±11.84)
*dnaJ*	3.78	−36.93 (±133.44)	**29.25 (±32.98)**	−5.97 (±29.17)	−82.4 (±38.27)	−0.61 (±12.8)	**98.58 (±1.38)**
*dnaK*	1.26	−189 (±25.15)	19.47 (±25.12)	5.93 (±26.75)	−87.37 (±30.17)	0.75 (±28.14)	−122.76 (±10.51)
*ecnB*	4.75	−207.51 (±26.82)	4.05 (±11.31)	**25.26 (±20.25)**	−86.37 (±25.34)	16.66 (±3.25)	**23.94 (±114.4**)
*fabH*	2.82	−30.08 (±142.87)	19.4 (±23.52)	3.76 (±25.18)	−88.74 (±38.58)	−9.42 (±32.18)	**46.18 (±68.28)**
*fimB*	3.78	−166.25 (±68.29)	17.69 (±33.13)	9.32 (±29.85)	−141.79 (±44.04)	−65.31 (±53.56)	**97.3 (±2.45)**
*gmhB*	3.93	−148.78 (±36.83)	**33.44 (±55.38)**	**55.39 (±16.57)**	−77.78 (±43.53)	**60.66 (±6.07)**	**97.42 (±2.21)**
*gntK*	4.51	−143.54 (±61.26)	16.33 (±22.69)	11.45 (±30.13)	−77.17 (±39.44)	−43.69 (±4.8)	**29.19 (±98.23)**
*holD*	3.79	−190.55 (±69.39)	**28.85 (±9.87)**	**40.55 (±42.29)**	−30.97 (±18.8)	13.3 (±22.79)	**76.81 (±44.43)**
*metR*	4.72	−119.01 (±73.02)	10.25 (±40.14)	**48.18 (±9.14)**	−45.78 (±44.83)	−4.28 (±27.65)	−88.81 (±48.94)
*narL*	4.91	−163.49 (±47.2)	**23.64 (±27.57)**	**62.32 (±14.41)**	−46.47 (±28.79)	6.47 (±26.48)	−115.65 (±22.38)
*nuoN*	6.03	−118.98 (±30.6)	**55.65 (±15.03)**	**41.32 (±15.03)**	−52.15 (±11.24)	4.87 (±14.32)	**20.86 (±104.77)**
*oxyR*	5.69	−164.37 (±75.84)	**62.53 (±15.66)**	**36.03 (±32.12)**	−45.03 (±34.09)	**27.51 (±23.22)**	2.42 (±61.69)
*pfkA*	3.80	−134.27 (±48.88)	**40.11 (±30.89)**	**57.22 (±8.21)**	−46.31 (±42.02)	12.18 (±21.63)	−75 (±57.62)
*pgi*	2.79	−113.64 (±45.57)	**37.64 (±16.31)**	**52.77 (±8.07)**	−41.07 (±10.14)	4.1 (±21.54)	**48.78 (±108.45)**
*pgm*	3.43	−140.32 (±84.01)	**38.95 (±15.56)**	**35.61 (±16.85)**	−59.53 (±36.21)	17.47 (±22.74)	**46.1 (±73.31)**
*phoQ*	3.87	−84.48 (±139.55)	**66.91 (±46.06)**	**48.66 (±12.1)**	−58.83 (±38.61)	−5.88 (±27.88)	**54.54 (±95.91)**
*ppc*	6.80	−154.47 (±169.15)	**44.06 (±25.28)**	**35.24 (±8.34)**	−90.37 (±45.68)	−39.37 (±37.86)	−210.34 (±77.81)
*priA*	5.45	−53.04 (±84.24)	7.59 (±60.1)	**36.27 (±6.14)**	−77.97 (±37.42)	−6.38 (±6.64)	**100.09 (±1.12)**
*purA*	5.54	−128.16 (±132.92)	−0.56 (±16.97)	−15.44 (±31.35)	−80.22 (±24.5)	−12.57 (±37.88)	**24.72 (±102.15)**
*pyrE*	4.74	−309.35 (±19.3)	2.3 (±32.84)	−27.4 (±37.32)	−76.66 (±107.67)	−75.19 (±47.46)	−7.77 (±151.65)
*rbfA*	2.86	**21.01 (±166.8)**	8.06 (±10.56)	**33.65 (±12.02)**	−82.54 (±58.02)	−15.16 (±34.11)	**97.41 (±2.85)**
*rfaC*	3.45	**97.14 (±1.12)**	15.54 (±14.47)	5.71 (±29.65)	−56.98 (±4.34)	7.27 (±23.69)	−87.13 (±9.31)
*rfaD*	2.89	−67.18 (±93.64)	**31.37 (±13.92)**	**48.52 (±10.63)**	−37.98 (±29.32)	**49.3 (±10.15)**	**57.13 (±91.19)**
*rfaE*	3.07	−128.04 (±21.89)	14.07 (±8.66)	**35.87 (±12.35)**	−72.34 (±50.57)	**25.47 (±17.14)**	**76.47 (±46.67)**
*rfaG*	3.31	−44.48 (±131.42)	**23.03 (±9.84)**	**36.55 (±16.5)**	−59.04 (±27.59)	−10.47 (±21.51)	**48.36 (±73.29)**
*rnt*	3.16	−149.17 (±62.64)	**40.77 (±20.82)**	**24.6 (±24.89)**	−56.45 (±93.29)	16.47 (±2.83)	−101.47 (±14.6)
*rpe*	6.17	−219.05 (±39.01)	**58.63 (±12.95)**	**50.89 (±11.9)**	−41.58 (±59.99)	−8.78 (±26.48)	−77.51 (±25.54)
*sstT*	5.27	−210.03 (±204.21)	−24.06 (±20.82)	**26.66 (±17.35)**	−138.41 (±60.75)	−49.4 (±38.47)	−123.18 (±68.15)
*surA*	3.89	−65.17 (±154.66)	−20.62 (±33.54)	**32.45 (±24.58)**	−138.14 (±59.38)	13.71 (±38.37)	**94.49 (±4.25)**
*thyA*	4.33	−136.19 (±42.18)	**60.58 (±30.63)**	**81.64 (±8.19)**	−29.87 (±27.49)	1.01 (±33.81)	**47.66 (±62.09)**
*tolA*	3.75	−205.09 (±40.21)	**28.77 (±17.16)**	**28.96 (±36.67)**	−8.43 (±96.09)	8.6 (±20.65)	**65.12 (±45.69)**
*tpiA*	3.00	−73.66 (±103.76)	**81.33 (±10.03)**	**64.24 (±10.77)**	−66.96 (±24.71)	−1.32 (±2.73)	**79.21 (±44.3)**
*truA*	3.13	−78.67 (±60.31)	**74.54 (±17.93)**	**33.11 (±16.65)**	−63.16 (±25.4)	2.47 (±4.11)	**97.34 (±0.83)**
*ubiC*	5.05	−68.31 (±161.13)	**43.08 (±23.62)**	**20.37 (±25.65)**	−70.02 (±14.93)	−22.36 (±24.72)	**97.74 (±2.09)**
*umuD*	4.35	−21.3 (±68.99)	72.87 (±16.18)	50.65 (±10.61)	−3.18 (±27.29)	−26.02 (±26.69)	66.74 (±69.81)
*ybaP*	3.67	−55.11 (±138.78)	**30.34 (±29.77)**	**24.66 (±9.9)**	−89.11 (±37.37)	−13.5 (±5.31)	**97.1 (±2.44)**
*yccM*	4.04	**62.93 (±70.19)**	**80 (±2.26)**	**31.49 (±14.63)**	−117.78 (±43.5)	−32.86 (±31.47)	**97.05 (±4.61)**
*ydcE*	5.00	−281.61 (±123.85)	**27 (±34.7)**	3.93 (±36.09)	−99.98 (±50.99)	−48.81 (±35.15)	−66.91 (±160.6)
*ydcX*	4.95	−217.59 (±32.43)	−4.93 (±12.79)	−11.21 (±34.76)	−32.58 (±88.8)	−31.43 (±35.46)	−6.77 (±105.28)
*ydeU*	3.93	−156.77 (±78.48)	−10.67 (±21.77)	7.5 (±20.75)	−99.88 (±51.27)	−41.07 (±37.79)	**77.3 (±43.22)**
*yegS*	3.71	**62.76 (±71.68)**	**45.29 (±24.26)**	**32.98 (±18.99)**	−112.6 (±89.9)	−15.79 (±28.14)	**77.42 (±43.28)**
*yfbB*	4.18	**70.57 (±52.43**)	0.41 (±20.9)	41.36 (±13.22)	−133.11 (±60.68)	−22.42 (±34.34)	**97.64 (±1.29)**
*yfeH*	4.94	−230.42 (±72.37)	−7.21 (±7.94)	−18.13 (±40.46)	−100.87 (±39.21)	−22.6 (±33.19)	**65.5 (±68.98)**
*yfgL*	5.24	−29.96 (±115.43)	**20.86 (±29.65)**	**35.42 (±13.97)**	−57.53 (±13.77)	−10.85 (±10.49)	**46.19 (±116.29)**
*ygfZ*	2.50	**21.51 (±164.65)**	−86.41 (±51)	−40.88 (±39.91)	−259.54 (±72.84)	−199.89 (±92.33)	−531.32 (±89.95)
*yheM*	3.00	−154.13 (±79.36)	13.39 (±31.54)	**34.44 (±24.65)**	−92.84 (±48.09)	−3.96 (±31.28)	−41.73 (±141.74)
*yhhH*	4.41	−83.13 (±128.4)	**56.35 (±28.8)**	**33.97 (±19.35)**	−48.01 (±34.47)	**22.42 (±8.68)**	−117.43 (±18.78)
*yigL*	4.28	−165.97 (±138.93)	−1.23 (±57.34)	**27.78 (±10.29)**	−167.66 (±80.66)	−53.8 (±37.53)	**48.89 (±109.08)**
*yihE*	4.58	−21.93 (±113.08)	**75.01 (±12.93)**	**44.39 (±13.07)**	−38.37 (±58.47)	8.52 (±3.91)	**44.24 (±109.43)**
*yjeK*	3.68	−48.06 (±133.73)	**26.68 (±37.56)**	**37.88 (±23.77)**	−81.49 (±23.54)	−3.65 (±36.66)	−130.9 (±19.59)
*yncA*	3.84	−146.47 (±72.89)	−28.03 (±33.87)	**23.27 (±16.8)**	−103.51 (±50.94)	−54.74 (±38.3)	**26.3 (±98.73)**
*ypjD*	2.68	−4.02 (±132.26)	7.18 (±32.02)	**30.29 (±13.93)**	−87.37 (±39.37)	−43.1 (±29.95)	**23.16 (±118.13)**

**Table 3:** The ρ-Spearman correlation coefficient between aBL and stressors. Statistically significant results for α = 0.05 are bolded. * the *p*-value is less than **0.05** (*p* < 0.05), *** the *p*-value is less than **0.001** (*p* < 0.001).

aBL	Single Stressors	Spearman’s ρ	*p*-Value	*p*-Value Significance
aBL sensitivity	H_2_O_2_	−0.450	0.0002	***
	Membrane stress	−0.310	0.0132	*
	•OH	−0.230	0.0698	
	NO•	0.099	0.4380	
	O_2_^−^	0.093	0.4630	
	Acidic pH	0.060	0.6380	

Correlograms are used to illustrate the correlation between aBL sensitivity and various stressors, including H_2_O_2_, O_2_^−^, NO•, acidic pH, membrane stress, and •OH. This visualization helps to assess how aBL sensitivity correlates with the growth defect of selected mutants induced by each stressor, if this is a positive or negative type of correlation. Such representations can facilitate the search for patterns and relationships, highlighting the interplay between the antimicrobial effects of aBL and single stressors. These insights are helpful in understanding the multifactorial effects of aBL and the potential mechanisms underlying microbial susceptibility under different stress conditions.

**Table 4:** The probability of sensitivity to stressor A subject to sensitivity to stressor B. The number of mutants sensitive to both selected stress factors is given in brackets.

Stressor A	Stressor B					
	H_2_O_2_	O_2_^−^	NO•	Acidic pH	Membrane Stress	•OH
H_2_O_2_	x	0.98 (40)	0.98 (46)	0.94 (58)	0.88 (23)	0.93 (57)
O_2_^−^	0.67 (40)	x	0.79 (37)	0.63 (39)	0.58 (15)	0.66 (40)
NO•	0.77 (46)	0.9 (37)	x	0.73 (45)	0.69 (18)	0.74 (45)
Acidic pH	0.97 (58)	0.95 (39)	0.96 (45)	x	0.96 (25)	0.97 (59)
Membrane stress	0.38 (23)	0.37 (15)	0.38 (18)	0.4 (25)	x	0.38 (23)
•OH	0.95 (57)	0.98 (40)	0.96 (45)	0.95 (59)	0.88 (23)	x

**Table 5:** Principal Component Analysis (PCA) analysis results. PC1 and PC2 represent the individual principal components independent from each other and capture the data’s maximum variance. R^2^ is the coefficient of determination.

	PC1		PC2	
Stressor	Factor Loading	R^2^	Factor Loading	R^2^
H_2_O_2_	0.15	0.02	0.66	0.44
O_2_^−^	0.51	0.26	−0.11	0.01
NO•	0.50	0.25	−0.06	0.00
Acidic pH	0.50	0.25	−0.23	0.05
Membrane stress	0.47	0.22	0.15	0.02
•OH	0.05	0.00	0.69	0.48

**Table 6:** The list of deleted genes in single-gene mutants of *E. coli* assigned to individual clusters.

Cluster 1	Cluster 2	Cluster 3
*atpC*	*atpA*	*atpD*
*atpG*	*atpB*	*dnaK*
*cydD*	*atpE*	*ecnB*
*dacA*	*atpF*	*fimB*
*deoB*	*atpH*	*gntK*
*gmhB*	*cpxA*	*ppc*
*holD*	*dnaJ*	*purA*
*metR*	*fabH*	*pyrE*
*narL*	*priA*	*sstT*
*nuoN*	*rbfA*	*ydcE*
*oxyR*	*rfaC*	*ydcX*
*pfkA*	*rfaE*	*ydeU*
*pgi*	*rfaG*	*yfeH*
*pgm*	*surA*	*yheM*
*phoQ*	*ubiC*	*yigL*
*rfaD*	*ybaP*	*yncA*
*rnt*	*yccM*	
*rpe*	*yegS*	
*thyA*	*yfbB*	
*tolA*	*yfgL*	
*tpiA*	*ypjD*	
*truA*		
*umuD*		
*yhhH*		
*yihE*		
*yjeK*		

**Table 7:** Median and interquartile range and the result of one-dimensional tests comparing these medians between each pair of clusters. ^1^ U Mann–Whitney Test with FDR correction. * the *p*-value is less than **0.05** (*p* < 0.05), ** the *p*-value is less than **0.01** (*p* < 0.01), *** the *p*-value is less than **0.001** (*p* < 0.001), **** the *p*-value is less than **0.0001** (*p* < 0.0001), ns not statistically significant.

	Group 1		Group 2			
Stressor	Cluster	Median (IQR)	Cluster	Median (IQR)	*p*-Value^1^	*p*-Value Significance ^1^
H_2_O_2_	1	−131.16 (−164.15, −79.79)	2	−29.96 (−55.11, 21.01)	0.0000	****
	1	−131.16 (−164.15, −79.79)	3	−177.62 (−219.96, −154.38)	0.0006	***
	2	−29.96 (−55.11, 21.01)	3	−177.62 (−219.96, −154.38)	0.0000	****
Membrane stress	1	5.67 (−3.02, 15.68)	2	−6.38 (−15.16, 13.71)	0.0740	ns
	1	5.67 (−3.02, 15.68)	3	−40.22 (−50.5, −10.42)	0.0000	****
	2	−6.38 (−15.16, 13.71)	3	−40.22 (−50.5, −10.42)	0.0020	**
NO•	1	48.35 (38.55, 54.74)	2	30.29 (8.39, 35.42)	0.0000	****
	1	48.35 (38.55, 54.74)	3	8.47 (0.15, 25.61)	0.0000	****
	2	30.29 (8.39, 35.42)	3	8.47 (0.15, 25.61)	0.0370	*
O_2_^−^	1	53.98 (31.89, 65.82)	2	15.54 (0.41, 30.34)	0.0000	****
	1	53.98 (31.89, 65.82)	3	3.18 (−5.5, 16.67)	0.0000	****
	2	15.54 (0.41, 30.34)	3	3.18 (−5.5, 16.67)	0.1080	ns
•OH	1	48.22 (−76.88, 70.33)	2	95.89 (76.47, 97.31)	0.0006	***
	1	48.22 (−76.88, 70.33)	3	8.59 (−56.05, 34.11)	0.2420	ns
	2	95.89 (76.47, 97.31)	3	8.59 (−56.05, 34.11)	0.0002	***
Acidic pH	1	−46.39 (−59.36, −38.08)	2	−81.65 (−89.11, −70.02)	0.0000	****
	1	−46.39 (−59.36, −38.08)	3	−91.61 (−101.53, −83.61)	0.0000	****
	2	−81.65 (−89.11, −70.02)	3	−91.61 (−101.53, −83.61)	0.0630	ns

**Table 8:** Key functional and biological significance across the clusters.

Cluster	Gene(s)	Function	Associated Pathway	Biological Significance
1	*atpC*, *atpG*, *nuoN*	ATP synthesis via oxidative phosphorylation	Energy metabolism	Key for ATP production and cellular energy homeostasis.
	*pfkA*, *pgi*, *pgm*, *rpe*, *tpiA*	Glycolysis, pentose phosphate pathway	Carbohydrate metabolism	Critical for energy production and biosynthetic precursors.
	*oxyR*, *narL*, *cydD*	Oxidative stress response	ROS detoxification, nitrogen metabolism	Protects cells under oxidative or nitrogen stress.
	*phoQ*, *metR*	Cellular adaptation, stress response	Phosphate and methionine regulation	Adaptation to nutrient limitations and environmental stress.
	*tolA*, *rfaD*	Membrane integrity, LPS synthesis	Membrane structure and protection	Essential for outer membrane stability and function.
2	*atpA*, *atpB*, *atpE*, *atpF*, *atpH*	F1Fo ATP synthase complex	Energy metabolism	Core to ATP generation and proton gradient maintenance.
	*cpxA*	Sensor for membrane stress	Two-component regulatory system	Activates stress response to misfolded membrane proteins.
	*dnaJ*	Molecular chaperone	Heat shock response	Repairs misfolded proteins and supports thermal adaptation.
	*fabH*	Fatty acid biosynthesis	Lipid metabolism	Initiates synthesis of membrane lipids, vital for adaptation.
3	*dnaK*, *fimB*	Protein folding, repair, adhesion regulation	Heat shock response, fimbriae assembly	Facilitates adaptation to stress and host interactions.
	*atpD*, *gntK*, *ppc*	Energy metabolism and intermediary pathways	Glycolysis, TCA cycle	Supports energy generation and carbon flux management.
	*purA*, *pyrE*	Nucleotide biosynthesis	Purine and pyrimidine metabolism	Provides precursors for DNA, RNA synthesis under stress.
	*yheM*, *yigL*, *ydeU*	Hypothetical	Unknown	Likely involved in environmental stress response and adaptation.

## Data Availability

The raw data supporting the conclusions of this article will be made available by the authors upon request.

## Supplementary Materials

The following supporting information can be downloaded at https://www.mdpi.com/article/10.3390/antiox13121583/s1.
