# Supplementary material for: Mimicking the Effects of Antimicrobial Blue Light: Exploring Single Stressors and Their Impact on Microbial Growth
Source: Antioxidants (Basel). 2024 Dec 23;13(12):1583. doi: 10.3390/antiox13121583 (PMC11673782; doi:10.3390/antiox13121583)
Supplement: Supplementary file 1 [file antioxidants-13-01583-s001.zip › antioxidants-3315014-supplementary.docx]

**Supplementary Materials:** *Mimicking the effects of antimicrobial blue light: exploring single stressors and their impact on microbial growth* by Beata Kruszewska-Naczk, Mariusz Grinholc and Aleksandra Rapacka-Zdonczyk

**Table S1**. The mean and standard deviation for each stressor.

| **Characteristic** | **N = 64**^1^ |
| --- | --- |
| aBL sensitivity | 3.91 (1.17) |
| H_2_O_2_ | -104 (91) |
| O_2_^-^ | 25 (32) |
| NO• | 29 (25) |
| Acid pH | -76 (40) |
| Membrane stress | -8 (36) |
| •OH | 17 (105) |
| ^1^Mean (SD) | |

**Table S2.** The number of mutants (n) for which the growth defect was higher than 20% and the mean growth defect for each stressor.

| Stressor | n | Mean growth defect |
| --- | --- | --- |
| H_2_O_2_ | 7 | 61.1 |
| Membrane stress | 9 | 35.12 |
| •OH | 45 | 41.58 |
| NO• | 36 | 47.1 |
| O_2_^-^ | 45 | 70.35 |
| Acid pH | 0 | - |


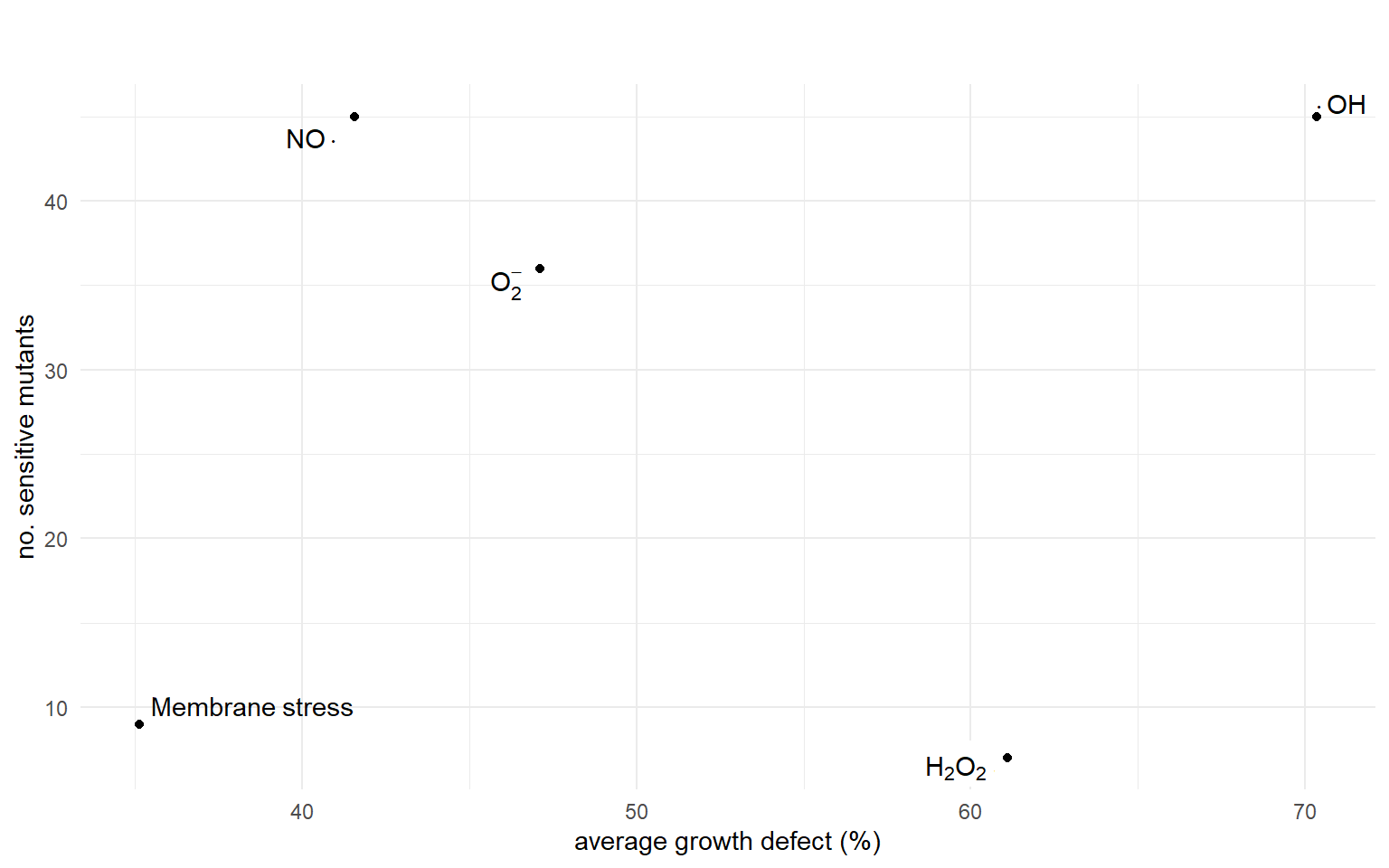


**Figure S1.** The count of knockout mutants displaying a growth defect, surpassing 20% against the mean growth deficiency of these strains.

**Table S3.** The number of mutants (n) for which the growth defect was smaller than -20% and the mean growth defect for each stressor.

| Stressor | n | Mean growth defect |
| --- | --- | --- |
| H_2_O_2_ | 53 | -133.16 |
| Membrane stress | 17 | -51.30 |
| •OH | 2 | -34.14 |
| NO• | 5 | -37.96 |
| O_2_^-^ | 16 | -129.16 |
| Acid pH | 62 | -77.75 |


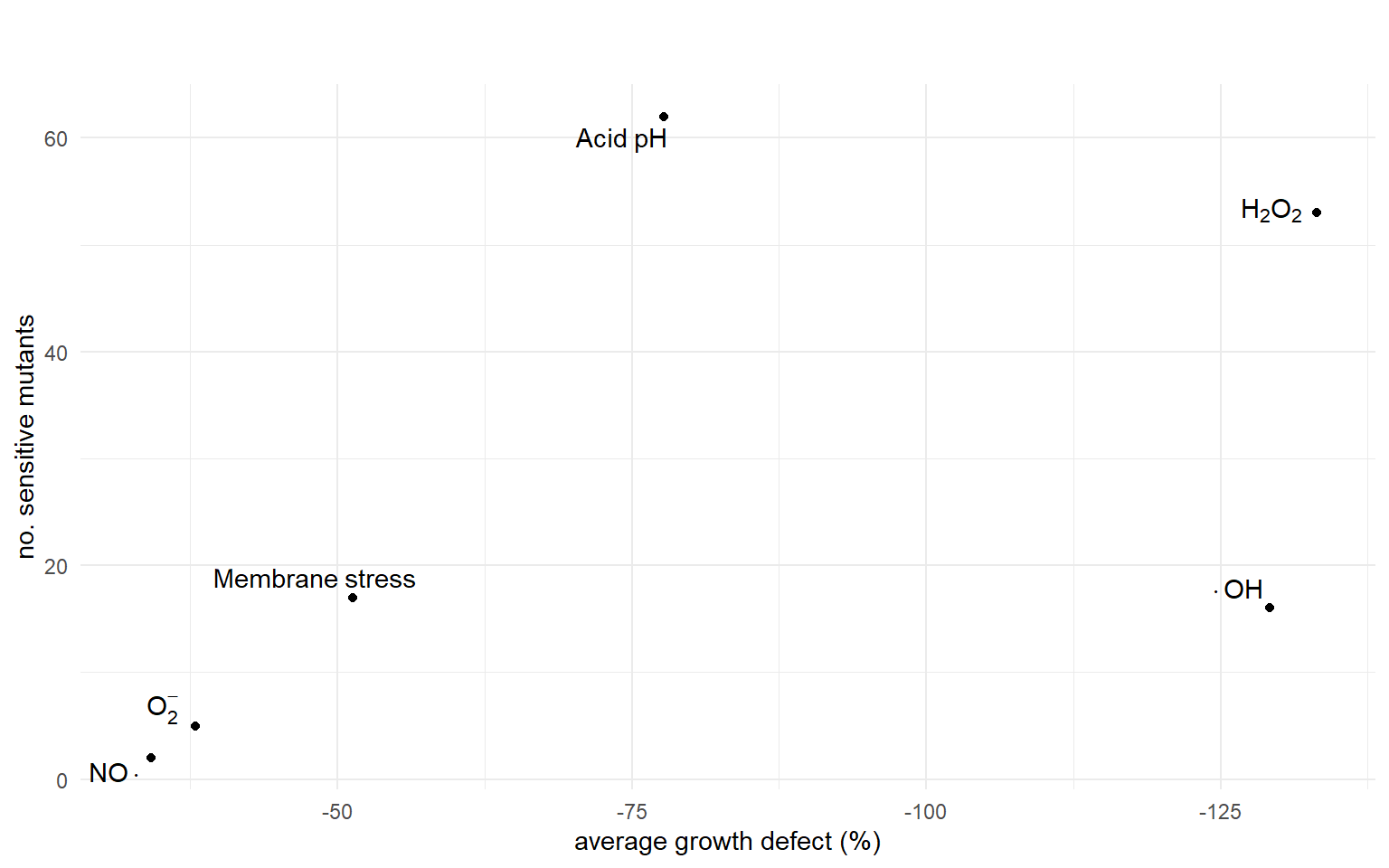


**Figure S2.** The count of knockout mutants displaying a growth defect smaller than -20% against the mean growth deficiency of these strains.


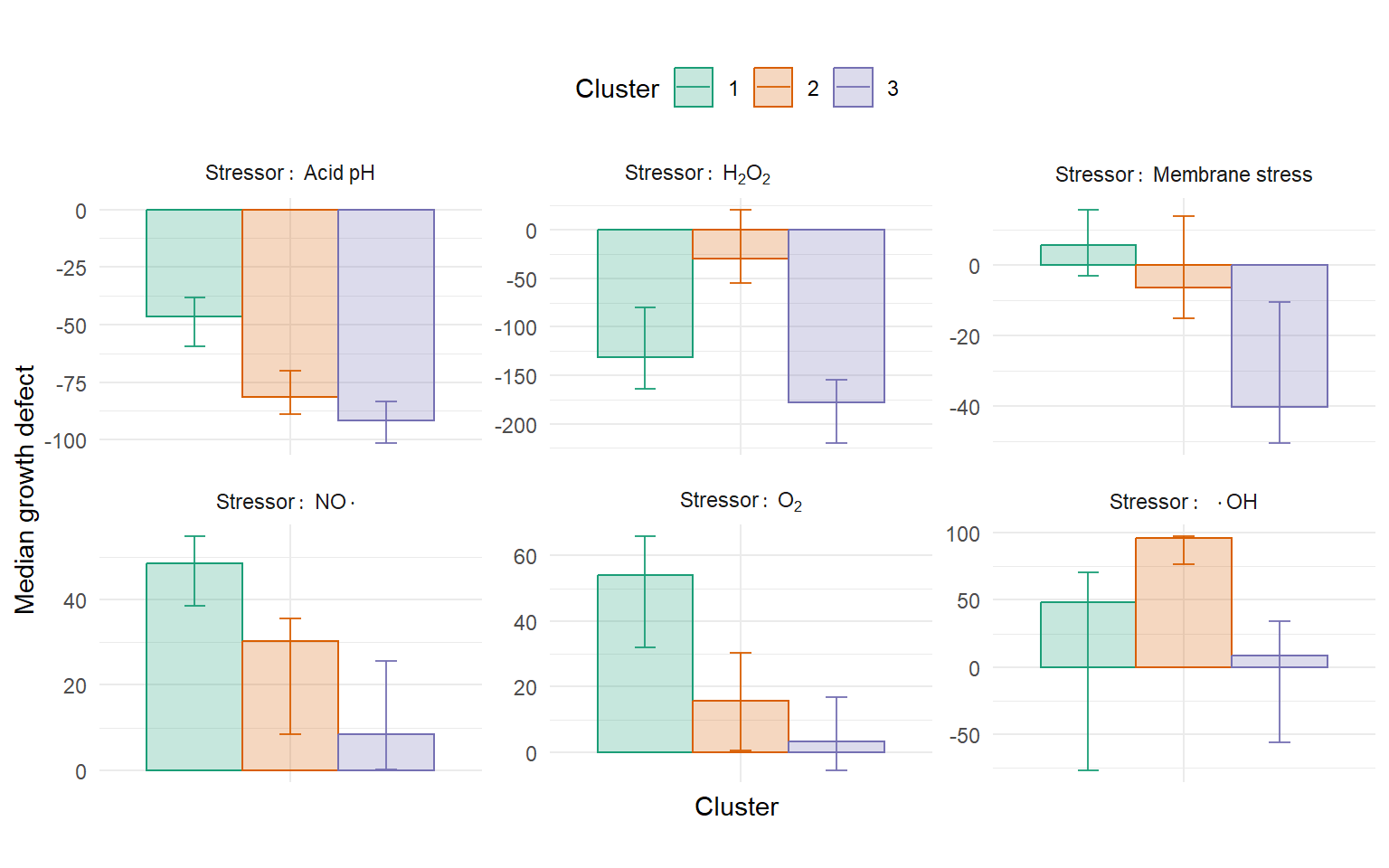


**Figure S3.** Median growth defect for clusters. A characterization of growth defect profiles for 3 clusters. The bars represents median values, while the error bars denote the Interquartile Range (IQS).


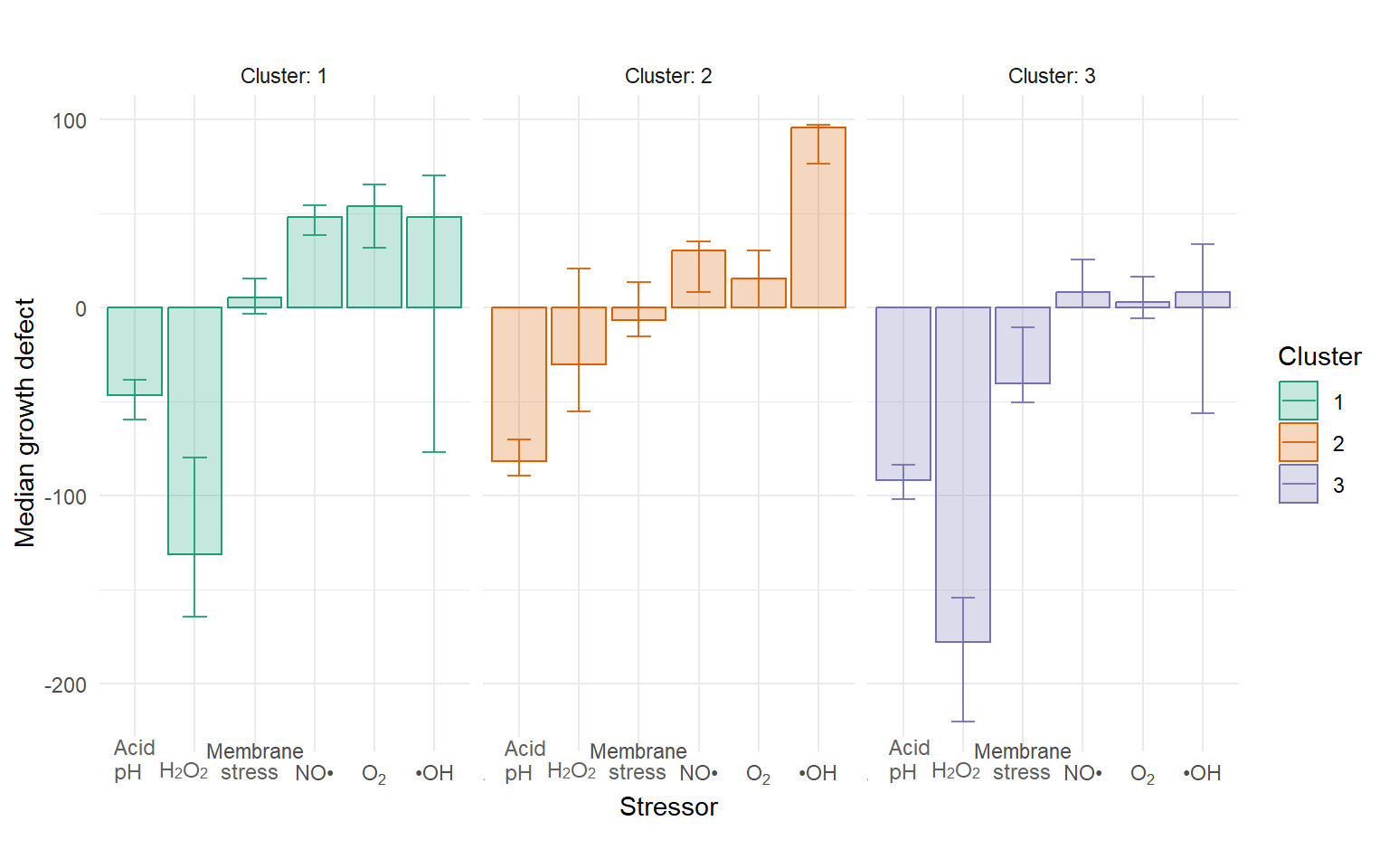


**Figure S4.** **Median growth defect for clusters.** A characterization of cluster profiles for each stressor. The bars represents median values, while the error bars denote the Interquartile Range (IQS).

**Table S4. List of aBL hypersensitive mutants with a description of the protein encoded by the deleted gene.**

| Lp. | Gene name | Synonyms | Description |
| --- | --- | --- | --- |
| 1. | *atpA* | *papA // uncA* | ATP synthase subunit alpha |
| 2. | *atpB* | *papD // uncB* | ATP synthase subunit a |
| 3. | *atpC* | *papG // uncC* | ATP synthase epsilon chain |
| 4. | *atpD* | *papB // uncD* | ATP synthase  subunit beta |
| 5. | *atpE* | *papH // uncE* | ATP synthase subunit c |
| 6. | *atpF* | *papF // uncF* | ATP synthase subunit b |
| 7. | *atpG* | *papC // uncG* | ATP synthase gamma chain |
| 8. | *atpH* | *papE // uncH* | ATP synthase subunit delta |
| 9. | *cpxA* | *rssE // ecfB // eup // ssd // ecf* | Sensor histidine kinase CpxA |
| 10. | *cydD* | *htrD* | ATP-binding/permease protein CydD |
| 11. | *dacA* | *pfv* | D-alanyl-D-alanine carboxypeptidase DacA |
| 12. | *deoB* | *tlr // drm // thyR* | Phosphopentomutase |
| 13. | *dnaJ* | *groP // grpC* | Chaperone protein DnaJ |
| 14. | *dnaK* | *groPF // groPC // seg // grpF // grpC // groPAB* | Chaperone protein DnaK |
| 15. | *ecnB* | *yjeU* | Entericidin B |
| 16. | *fabH* |  | 3-oxoacyl-[acyl-carrier-protein] synthase 3 |
| 17. | *fimB* | *pil* | Type 1 fimbriae regulatory protein FimB |
| 18. | *gmhB* | *yaeD* | D-glycero-beta-D-manno-heptose-1,7-bisphosphate 7-phosphatase |
| 19. | *gntK* |  | Thermoresistant gluconokinase |
| 20. | *hldD* | *yqiF // rfaE // gmhC // waaE* | ADP-L-glycero-D-manno-heptose-6-epimerase |
| 21. | *holD* |  | DNA polymerase III subunit psi |
| 22. | *metR* |  | HTH-type transcriptional regulator MetR |
| 23. | *narL* | *frdR // narR* | Nitrate/nitrite response regulator protein NarL |
| 24. | *nuoN* |  | NADH-quinone oxidoreductase subunit N |
| 25. | *oxyR* | *momR // mor* | Hydrogen peroxide-inducible genes activator |
| 26. | *pfkA* |  | ATP-dependent 6-phosphofructokinase isozyme 1 |
| 27. | *pgi* |  | Glucose-6-phosphate isomerase |
| 28. | *pgm* | *gpmA //blu* | 2,3-bisphosphoglycerate-dependent phosphoglycerate mutase |
| 29. | *phoQ* |  | Sensor protein PhoQ |
| 30. | *ppc* | *asp // glu* | Coenzyme A biosynthesis bifunctional protein CoaBC |
| 31. | *priA* | *srgA* | Primosomal protein N' |
| 32. | *purA* | *adeK* | Adenylosuccinate synthetase |
| 33. | *pyrE* |  | Orotate phosphoribosyltransferase |
| 34. | *rbfA* | *sdr-43 // yhbB* | 30S ribosome-binding factor |
| 35. | *rfaC* | *rfa-2 // waaC // yibC* | Lipopolysaccharide heptosyltransferase 1 |
| 36. | *rfaE* | *hldE // yqiF // gmhC // waaE* | Bifunctional protein HldE |
| 37. | *rfaG* | *waaG* | Lipopolysaccharide core biosynthesis protein RfaG |
| 38. | *rnt* |  | Ribonuclease T |
| 39. | *rpe* | *dod // yhfD* | Ribulose-phosphate 3-epimerase |
| 40. | *sstT* | *ygjU* | Serine/threonine transporter SstT |
| 41. | *surA rfaD* | *waaD // hldD // htrM // nbsB* | Chaperone SurA |
| 42. | *thyA* |  | Thymidylate synthase |
| 43. | *tolA* | *cim // excC // lky // tol-2* | Tol-Pal system protein TolA |
| 44. | *tpiA* | *tpi* | Triosephosphate isomerase |
| 45. | *truA* | *asuC // hisT // leuK* | tRNA pseudouridine synthase A |
| 46. | *ubiC* |  | Chorismate pyruvate-lyase |
| 47. | *umuD* |  | Protein UmuD |
| 48. | *ybaP* |  | TraB family protein YbaP |
| 49. | *yccM* |  | Putative electron transport protein YccM |
| 50. | *ydcE* | *pptA* | Tautomerase PptA |
| 51. | *ydcx* | *ortT* | Orphan toxin OrtT |
| 52. | *ydeU* | *orfT // ydeK // ydeU // b1509 // b1510 // ECK1502* | AIDA-I family autotransporter YneO |
| 53. | *yegS* |  | Lipid kinase YegS |
| 54. | *yfbB* | *menH* | 2-succinyl-6-hydroxy-2,4-cyclohexadiene-1-carboxylate synthase |
| 55. | *yfeH* |  | Putative symporter YfeH |
| 56. | *yfgL* | *bamB* | Outer membrane protein assembly factor BamB |
| 57. | *ygfZ* | *yzzW* | tRNA-modifying protein YgfZ |
| 58. | *yheM* | *tusC* | Protein TusC |
| 59. | *yhhH* |  | PF15631 family protein YhhH |
| 60. | *yigL* |  | Pyridoxal phosphate phosphatase YigL |
| 61. | *yihE* | *orfA // srkA* | Stress response kinase A |
| 62. | *yjeK* | *epmB* | L-lysine 2,3-aminomutase |
| 63. | *yncA* | *mnaT* | L-amino acid N-acyltransferase MnaT |
| 64. | *ypjD* |  | Inner membrane protein YpjD |
